# Molecular Basis of Differential Sensitivity of Myeloma Cells to Clinically Relevant Bolus Treatment with Bortezomib

**DOI:** 10.1371/journal.pone.0056132

**Published:** 2013-02-27

**Authors:** Tamer B. Shabaneh, Sondra L. Downey, Ayrton L. Goddard, Michael Screen, Marcella M. Lucas, Alan Eastman, Alexei F. Kisselev

**Affiliations:** 1 Norris Cotton Cancer Center, The Geisel School of Medicine at Dartmouth, Lebanon, New Hampshire, United States of America; 2 Department of Pharmacology and Toxicology, The Geisel School of Medicine at Dartmouth, Lebanon, New Hampshire, United States of America; 3 Department of Biology & Biochemistry, University of Bath, Bath, United Kingdom; The Ohio State University, United States of America

## Abstract

The proteasome inhibitor bortezomib (Velcade) is prescribed for the treatment of multiple myeloma. Clinically achievable concentrations of bortezomib cause less than 85% inhibition of the chymotrypsin-like activity of the proteasome, but little attention has been paid as to whether in vitro studies are representative of this level of inhibition. Patients receive bortezomib as an intravenous or subcutaneous bolus injection, resulting in maximum proteasome inhibition within one hour followed by a gradual recovery of activity. In contrast, most in vitro studies use continuous treatment so that activity never recovers. Replacing continuous treatment with 1 h-pulse treatment increases differences in sensitivity in a panel of 7 multiple myeloma cell lines from 5.3-fold to 18-fold, and reveals that the more sensitive cell lines undergo apoptosis at faster rates. Clinically achievable inhibition of active sites was sufficient to induce cytotoxicity only in one cell line. At concentrations of bortezomib that produced similar inhibition of peptidase activities a different extent of inhibition of protein degradation was observed, providing an explanation for the differential sensitivity. The amount of protein degraded per number of active proteasomes correlated with sensitivity to bortezomib. Thus, (i) in vitro studies of proteasome inhibitors should be conducted at pharmacologically achievable concentrations and duration of treatment; (ii) a similar level of inhibition of active sites results in a different extent of inhibition of protein breakdown in different cell lines, and hence a difference in sensitivity.

## Introduction

The proteasome inhibitor bortezomib (Velcade, PS-341) is prescribed for the treatment of multiple myeloma and mantle cell lymphoma. The second proteasome inhibitor, carfilzomib (Kyprolis, PR-171) [1], was recently approved by the FDA for the treatment of relapsing and refractory myeloma. At least four novel proteasome inhibitors — marizomib (salinosporamide A, NPI-0052) [2], CEP-18770 [3], MLN-9708 [4], and PR-047 [5]—are at different stages of clinical development.

Bortezomib is highly cytotoxic to all multiple myeloma cell lines in vitro [6], but in vivo only ∼40% of myeloma patients respond to this drug given as a single agent [7]. One reason for this discrepancy may be that in vitro concentrations of bortezomib and length of exposure to this agent exceed those that can be achieved in vivo at the maximal tolerated dose (MTD). Most studies of bortezomib in cell culture have utilized continuous incubation for 24–48 h. In the clinical setting, patients receive intravenous or subcutaneous bolus injections twice weekly. When bortezomib is injected intravenously at the MTD, the blood plasma concentration peaks at 100–200 ng/mL (260–520 nM) 5 minutes after IV injection followed by rapid decrease [8]. Subcutaneous injection results in ∼10-fold lower maximal concentration, which is achieved 30 minutes after injection. The maximal concentration of the drug is maintained for 1–2 h so that total exposure to the drug (area under the Drug concentration-Time curve) is the same as after iv administration. Efficacy of the agent does not depend on the administration route [9].

The primary target of botezomib is the chymotrypsin-like activity of the proteasome. At the MTD, the mean maximal inhibition of proteasome in patient's peripheral blood cells is 73% after the first dose and up to 83% after subsequent doses [8], [10]–[13]. This inhibition is achieved within 5–30 min of administration. The inhibition stays at this level for ∼1 h and then slowly decreases to 0–25% 48–72 hours later [8]–[11], [14]. When bortezomib is administered subcutaneously maximal proteasome inhibition in blood is 5% lower than after IV dose and is achieved 2 h after administration. However, the rate of recovery is slightly slower and the area under the effect-time curve is the same as after intravenous administration [9].

Proteasome inhibition inside MM tumors in patients has not been studied. In a few clinical cases analyzed, inhibition of proteasome in solid tumor biopsies was found to be the same as in blood [10], [11]; however, inhibition in bone marrow was found to be half of the inhibition in blood of the same patient [11]. Proteasome inhibition in xenograft tumors in mice was ∼1/2 of the inhibition in blood [15]. Hence we should assume that inhibition of the proteasome inside myeloma tumors does not exceed and most likely is even lower than in blood.

The proteasome core has three pairs of active sites for proteolysis – chymotrypsin-like (ß5), trypsin-like (ß2), and caspase-like (ß1). Cells and tissues of the immune system, including multiple myeloma cells, also contain immunoproteasomes, which express different active-site subunits, i.e., ß5i, ß2i, and ß1i. Chymotrypsin-like sites (ß5, ß5i) are the primary targets of bortezomib, but ß1, ß1i, and ß2i are also inhibited, albeit with lesser potency, and ß2 is activated [16]–[18]. Inhibition of the proteasome in blood of patients is evaluated based on cumulative inhibition of ß5 and ß5i (chymotrypsin-like) sites.

Although the chymotrypsin-like sites are the most important in protein degradation, our earlier work has indicated that specific inhibition of these sites is not sufficient to block protein breakdown, and co-inhibition of either caspase-like or trypsin-like sites is required [17]. This raises the question of how much protein degradation is blocked at clinically achievable levels of proteasome inhibition, when chymotrypsin-likes sites are inhibited by not more than 85%. Inhibition of caspase-like sites and trypsin-like sites has not been reported in clinical samples; but, based on data in multiple myeloma cell lines, we predict that, at the MTD of bortezomib, caspase-like activity is partially inhibited and trypsin-like sites may even be activated. Effects of bortezomib on protein degradation in multiple myeloma cells have not been reported in the literature.

It has been suggested that myeloma cells are particularly sensitive to proteasome inhibitors due to their high ratio of proteasome workload to proteasome expression levels (the “load/capacity” hypothesis) [19]. Myeloma cells produce large amounts of immunoglobulins, complex four-chain molecules with several inter- and intra-chain disulfide bridges. Polypeptide chains that fail to fold or assemble have to be degraded via the endoplasmic reticulum (ER)-degradation pathway, imposing a heavy load on the proteasomes of these cells. Consistent with this hypothesis, increased production of immunoglobulins sensitizes myeloma cells to proteasome inhibitors [20].

In this study, we have compared the effects of continuous bortezomib treatment with a 1-h pulse on a panel of multiple myeloma cell lines. We observed that this shorter incubation increased the differences in sensitivity between cell lines. The most sensitive cell lines underwent apoptosis at clinically achievable bortezomib concentrations and at faster rates than the least sensitive ones; the least sensitive lines remained 100% viable under these conditions. We then found that similar inhibition of active sites by bortezomib causes stronger inhibition of protein breakdown in sensitive cell lines, potentially explaining their enhanced sensitivity.

## Experimental Procedures

### Cell lines, inhibitors and antibodies

**Figure 1:**
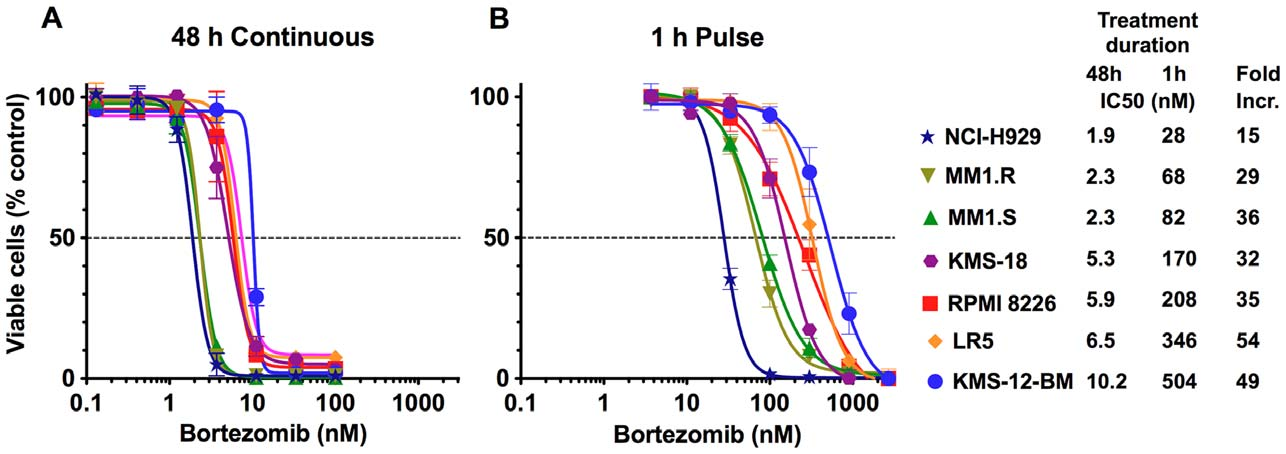
Comparison of 48-h continuous and a 1-h treatment of multiple myeloma cells with bortezomib. A. Cells were treated with bortezomib for 48 h, and then assayed for viable cells with the Alamar Blue mitochondrial dye conversion assay. Mock-treated cells served as control. Values are means±S.E.M of two experiments. B. Cells were treated with bortezomib for 1 h, then cultured in a drug-free media for an additional 47 h and finally assayed for viable cells with Alamar blue. Values are mean ± S.E.M of 4–13 measurements. IC_50_ values are presented in the legend.

**Figure 2:**
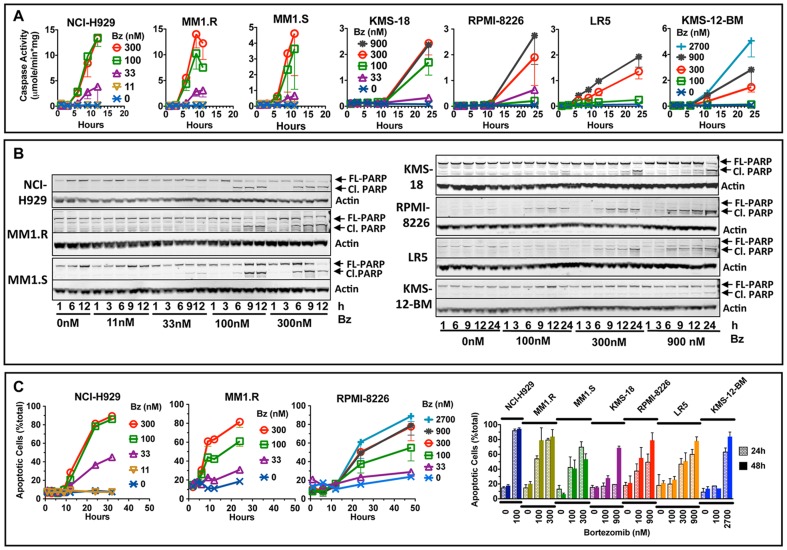
Bortezomib-treated multiple myeloma cells undergo apoptosis at different rates. Caspase-3 activity (A) and cleavage of PARP (B) were measured in extracts of cells treated with bortezomib for 1 h, and then cultured in drug-free media. B. Cleavage of PARP was assessed by western blotting. C. The % of Annexin V-positive cells was determined by flow cytometry. Cells on the first 3 graphs were analyzed 24 h after the start of 1-h bortezomib treatment. Values are averages ± S.E.M of 2–4 independent experiments.

NCI-H929 and RPMI-8226 cells were obtained from American Tissue Culture Collection. KMS-12-BM [21], and KMS-18 [22] cell lines were provided by Takemi Otsuki (Kawasaki Medical School, Japan). The MM1.S cell line and its dexamethasone-resistant derivative MM1.R [23] were provided by Dr. Steven Rosen (Northwestern University, Chicago, IL). Sensitivity to dexamethasone was verified with Alamar Blue assay. RPMI-8226 derived melphalan-resistant LR5 cells [24] were provided by Dr. William Dalton (Moffit Cancer Center, Tampa, FL). All cell lines were cultured in RPMI-1640 media supplemented with 10% fetal bovine serum (FBS), penicillin (100 µg/ml), streptomycin (100 units/ml), and anti-mycoplasma antibiotic plasmocin (2.5 µg/mL, Invivogen, San Diego CA). Bortezomib was purchased from Milleneum (Cambridge, MA) through Dartmouth-Hitchcock Medical Center pharmacy (experiments on Fig. 1, 2C) or LC laboratories (all other data). Drug from both sources had the same effect on cell viability in experiments shown in Fig. 1B. Benzyloxycarbonyl-Leu-Leu-Leu-epoxyketone (ZL3ek) was synthesized as described [25]. Poly-ADP-ribose polymerase (PARP) antibodies were from Cell Signaling (catalog # 9542) and actin antibodies were from Abcam (catalog # AB3280).

### Cell viability, apoptosis, total protein and proteasome assays

Viable cells were assessed with Alamar Blue (AbD Serotec or Invitrogen) as described [26]. Annexin V staining was performed using a Guava Nexin kit followed by analysis on a Guava mini flow cytometer. Caspase-3 and -7 activities were measured in extracts of cells lysed with digitonin lysis buffer (DLB, 50 mM Tris, pH 7.5, 250 mM sucrose, 5 mM MgCl_2_, 1 mM ATP, 0.5 mM EDTA, 1 mM DTT, 0.025% digitonin) using Ac-DEVD-7-amido-4-methylcoumarinamide (amc) substrate as described [26]. For western blot analysis cells were lysed in whole cell lysis buffer (50 mM Tris, pH 7.5, 10% glycerol, 5 mM MgCl_2_, 1 mM EDTA, and 0.5% CHAPS). Total protein was determined using Pierce 660 nM Protein assay. The activity of each proteasome active site in cells was determined using site-specific luminogenic substrates Suc-LLVY-amino-luciferine (aLuc), Z-nLPnLD-aLuc, and Boc-LRR-aLuc (ProteasomeGlo assay, Promega). The specificity of this assay in multiple myeloma cells has been established previously [26], [27].

### Measurement of protein synthesis and degradation rates

5×10^5^ cells (three replicates for each condition) were seeded in suspension cultures in RPMI-1640 media containing half-normal concentration of Leu and supplemented with 10 µCi/mL [^3^H]Leu and 10% dialyzed FBS. For synthesis measurements, 200 µL aliquots of cultures were withdrawn and mixed with 1/10 volume of ice-cold 100% trichloroacetic acid (TCA) in pre-cooled tubes. After 25 min incubation on ice, samples were centrifuged for 15 min at 20,000 g. Pellets of TCA-precipitated proteins were washed twice with ice-cold acetone, air-dried, and dissolved in 20 µL of 100% TCA. 20 µL of the supernatants from the 10% TCA precipitation step and 100% TCA-dissolved pellets were mixed with 250 µL scintillation fluid and counted on a scintillation plate reader. The amount of incorporated [^3^H]Leu was calculated as a percentage of total radioactivity in the culture, and replicates were averaged and plotted against time. Protein synthesis rates were calculated from the slopes of the curves. For synthesis of short-lived proteins, [^3^H]Leu incorporation was measured over a 1-h period. For synthesis of long-lived proteins, [^3^H]Leu incorporation was measured over a 16-h period.

To measure the breakdown of short-lived proteins, cultures of MM cells (5×10^5^ cells/ml) were pulse-labeled with 10 µCi/mL [^3^H]Leu for 1 h, and then washed 3 times with warm chase media (RPMI-1640 media containing Leu at 2.5× normal concentration (1 mM)). (Cells were washed four times during optimization of the protocol; the fourth wash was found to contain background counts—Table S1.) After washing, each suspension culture was incubated in the chase media and after 1 h, treated with TCA, and analyzed by scintillation counting as in the protein synthesis experiment. The calculations were performed as follows: percentage protein breakdown = total dpm in TCA supernatant/(total dpm in TCA supernatant+total dpm in TCA pellet)×100%. Replicates were averaged. Treatment with ZL3ek and bortezomib was performed during pulse labeling. These inhibitors did not affect protein synthesis rates (Table S2). ZL3ek is an irreversible epoxyketone inhibitor that forms two stable covalent bonds with the proteasome catalytic threonine [28] thereby preventing recovery during the chase.

Long-lived proteins in MM cells were radiolabeled under the same conditions used for short-lived proteins, except that duration of labeling was 16 h. Cells were washed three times with chase media, and incubated in the chase media for 1 h to allow for the degradation of short-lived proteins. Treatment with ZL3ek (10 µM) was performed during this 1 h incubation. After an additional wash with chase media to remove the inhibitor, cells were resuspended in fresh chase media. Aliquots of culture were withdrawn at 0, 1, 2 h and treated with TCA as described in protein synthesis experiments. % Protein degraded was calculated as (total cpm in the supernatant×100%)/(total cpm in pellet+total cpm in the supernatant) and plotted against time. The rate of degradation of long-lived proteins (%/h) was determined as the slope of resulting linear regression.

## Results

### Comparison of cytotoxicity following 1 h and 48 h incubation

In the initial experiment, seven myeloma cell lines were incubated with bortezomib continuously for 48 h, after which viable cells were assessed with Alamar Blue (Fig. 1A). All lines were highly sensitive, with an IC_50_ between 1.9 and 10.2 nM. When incubation was shortened to 1 h, followed by a subsequent 47 h incubation in drug-free media, the IC_50_ in all cells increased but the magnitude of the increase varied 15–54-fold, so that the difference in IC_50_ across the panel increased to 18-fold, from 28 to 504 nM (Fig. 1B). This 1-h exposure of cells to bortezomib resembles the brief exposure of cells to the maximal concentration of the agent when it is administered subcutaneously [9]. The majority of IC_50_ values are higher than ∼50 nM (20 ng/mL), which is the maximal concentration of the drug achieved in blood after subcutaneous administration [9]. The biggest difference in cytotoxicity was observed upon 1-h treatment with 100 nM bortezomib, which was highly cytotoxic to NCI-H929 cells but produced only a slight decrease in the number of viable LR5 and KMS-12-BM cells. Thus, shortening the treatment time to a more clinically relevant 1 h reveals larger differences in multiple myeloma cell sensitivity to bortezomib than previously appreciated.

In addition, we observed that the sensitive cell lines underwent apoptosis at faster rates following a 1-h treatment with bortezomib (Fig. 2). Caspase activation (Fig. 2A) and PARP cleavage (Fig. 2B) were observed in NCI-H929, MM1.R and MM1.S cells by 6 h, while it was not detected in KMS-12-BM and KMS-18 cells until 12 h or even 24 h after the treatment, even at the higher concentrations required to induce apoptosis in these cell lines (Fig. 2A and B). In RPMI-8226 and LR5 cell lines, only traces of caspase activity were detected 6 h after treatment. Similar results were obtained when apoptotic cells were quantified with Annexin V+ and analyzed by flow cytometry (Fig. 2C).

### Clinically achievable proteasome inhibition is not sufficient to induce apoptosis in the majority of myeloma cell lines

**Figure 3:**
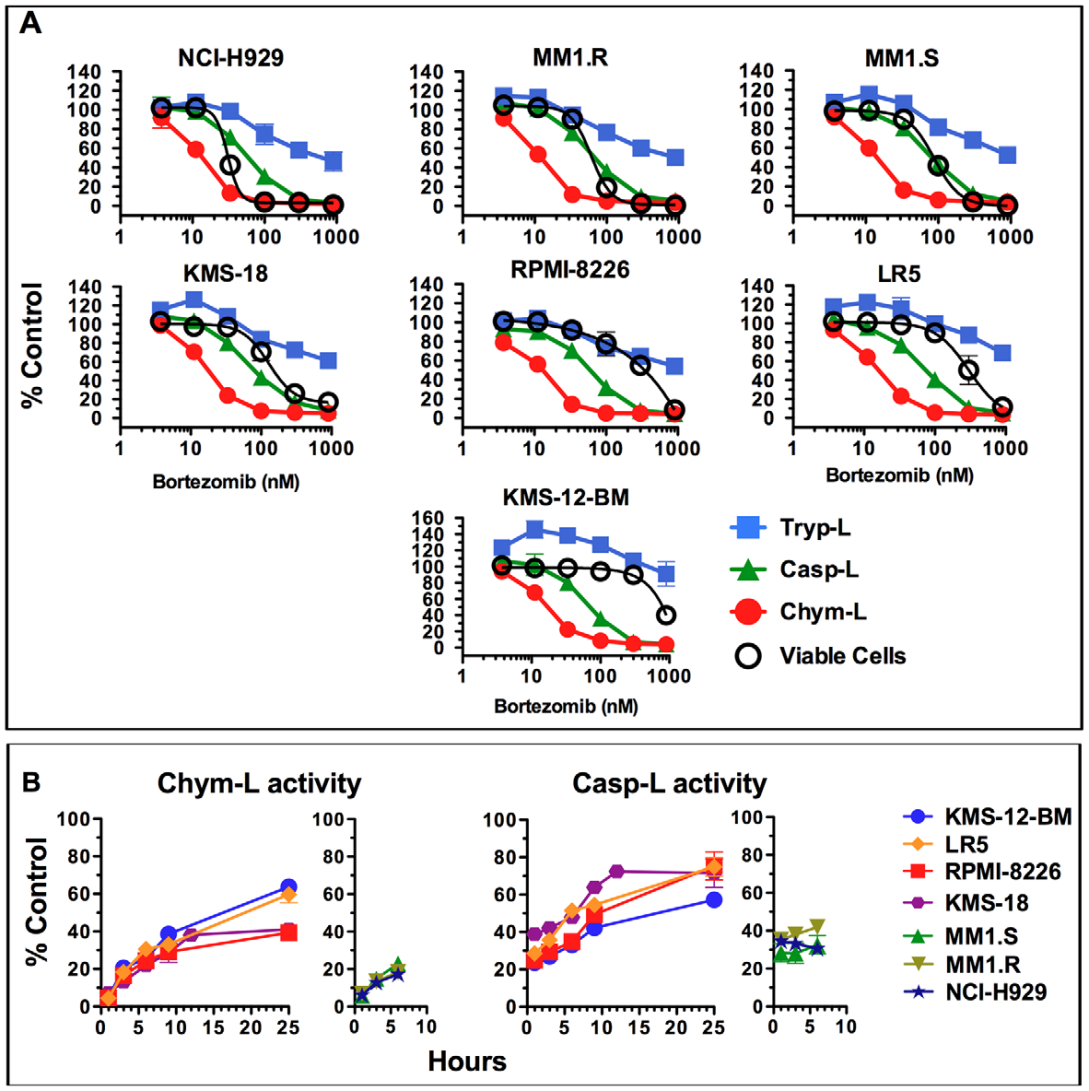
One-hour treatment with bortezomib causes similar inhibition of proteasome inside multiple myeloma cells. A. Inhibition of active sites was measured in cells immediately after 1-h of treatment with bortezomib. Mock-treated cells served as control. An aliquot of cells was cultured in fresh drug-free media for an additional 48 h, followed by Alamar Blue assay for cell viability. Values are averages±S.E.M of 2 or 3 experiments. The % of viable cells differ from Fig. 1B where they are averages of more repeats. B. Recovery of activity in cells treated for 1 h with 100 nM bortezomib. The first measurement was immediately after removal of bortezomib. Values are averages ± S.E.M of 2 independent experiments. Mock-treated cells served as controls. The activity is normalized to cell count at time zero. In NCI-H929, MM1.R and MM1.S cells, no data is presented at time points beyond 6 h because the number of viable cells decreases rapidly due to cell death (Fig. 2). Western blots analyzing proteasome amounts are shown on Fig. S1.

We next asked whether cytotoxicity is observed upon incubation with bortezomib at concentrations that result in maximal clinically achievable levels of proteasome inhibition (i.e., 70–85% inhibition of the proteasome's chymotrypsin-like sites). For this purpose, residual activity of proteolytic sites was measured immediately after a 1-h incubation with bortezomib and plotted against the number of viable cells after a further 47-h incubation in drug-free media (Fig. 3A). 85% inhibition of the chymotrypsin-like sites was achieved at 33 nM bortezomib in all cell lines. This concentration is slightly lower than the maximal concentration in blood plasma of MM patients treated subcutaneously. In the majority of cell lines little or no reduction of viable cells (Fig. 3A) and caspase activation (Fig. 2A) was observed at this concentration. The NCI-H929 cell line was the only exception as a clear reduction in viable cells was observed as the bortezomib concentration increased from 11 to 33 nM (Fig. 3A). In the majority of cell lines, >95% inhibition of chymotrypsin-like sites and co-inhibition of caspase-like sites was needed to reduce the number of viable cells to below 10%. This indicates that most MM cell lines are resistant to clinically achievable levels of proteasome inhibition.

### Differences in recovery of proteasome activity cannot explain differential sensitivity

We next set out to determine the molecular basis of the differences in sensitivity to bortezomib. As the levels of proteasome inhibition in all cell lines were similar, the observed differences in cell sensitivity to bortezomib cannot be attributed to different cell permeability of the drug, drug efflux, or a mutation in the proteasome-binding site.

Theoretically, bortezomib is a reversible inhibitor. Therefore, another reason for differential sensitivity could be different rates of recovery of activity after removal of the drug. To determine whether this is the case, we measured activity of the chymotrypsin and caspase-like sites at different times following 1-h treatment with 100 nM bortezomib, a concentration that generated largest differences in sensitivity (Fig. 3B). During the first six hours, 11–26% of chymotrypsin-like activity and −4–23% caspase-like activity recovered. Recovery continued in the cell lines that were not undergoing apoptosis. This rate of recovery of chymotrypsin-like activity is similar to the rate of recovery in blood of bortezomib-treated patients [10], [12]. Since the largest differences are observed at time points after death is initiated in sensitive lines (NCI-H929, MM1.R, MM1.S), we conclude that the difference in recovery of activity during drug washout cannot explain all the differences in sensitivity.

The moderate recovery observed in these experiments could be due to the synthesis of new proteasomes [29]. We used western blots to test whether the amount of proteasome subunits increase in bortezomib-treated RPMI-8226, LR5 and KMS-12-BM cells but did not observe an increase (Fig. S1). Thus, synthesis of new proteasome can account for recovery of activity only if inhibited proteasomes are degraded. It is not known whether inhibited proteasomes undergo selective degradation. Therefore, the most likely mechanism of recovery is dissociation of the drug.

### Effects of bortezomib on protein breakdown in MM cells

We next tested whether differences in inhibition of protein turnover could account for differences in sensitivity to bortezomib. Our earlier studies had established that both chymotrypsin-like sites and either caspase-like or trypsin-like sites have to be inhibited to achieve significant inhibition of protein degradation [17]. The largest differences in sensitivity were observed at 100 nM bortezomib, when caspase-like sites were only partially inhibited (Fig. 3), suggesting that the ability of proteasomes to degrade proteins was not completely impaired. Using pulse-chase experiments to measure overall protein degradation, we found that 23–30% of proteins labeled with [^3^H]Leu during a 1-h pulse were degraded during the first hour of the chase (Table 1). Although we did not determine the composition of this group of proteasome substrates, we assume that abnormal, misfolded proteins, predominantly immunoglobulin light and/or heavy chains, constitute the bulk of these short-lived proteins.

**Table 1:** Effect of inhibitors on degradation of short-lived proteins in multiple myeloma cells.

Cell line	Total degradation	Inhibitor	(µM)	Inhibition of Total Degradation		Inhibition of Proteasomal Degradation	Viable Cells
	(%/h)			(%)	(n)	(%)	(%)
NCI-H929	30.0	ZL3ek	10	61±3	5		
		bortezomib	0.1	34±3	2	55	3
MM1.R	26.3	ZL3ek	10	60±4	4		
		bortezomib	0.1	29±1	2	48	32
MM1.S	26.1	ZL3ek		70±4	2		
		bortezomib	0.1	30±5	2	43	44
KMS-18	26.3	ZL3ek	10	62±4	4		
		bortezomib	0.1	23±4	2	37	77
		bortezomib	0.9	53±2	3	86	12
RPMI-8226	26.2	ZL3ek	10	62±1	3		
		bortezomib	0.1	16±3	2	26	72
		bortezomib	0.9	39±5	3	57	11
LR5	23.2	ZL3ek	10	73±2	2		
		bortezomib	0.1	17±2	4	23	95
		bortezomib	0.9	35±3	3	48	13
KMS-12-BM	25.7	ZL3ek	10	54±4	4		
		bortezomib	0.1	16±1	3	30	95
		bortezomib	2.7	37±2	4	69	6

Degradation of short-lived proteins was analyzed after 1-h pulse labeling with [^3^H]Leu followed by 1-h chase. Total degradation was calculated as % of [^3^H]Leu incorporated during released in the TCA-soluble fraction during 1 h chase. Treatment with inhibitors was performed during pulse labeling.

% inhibition of degradation (at 1 h) was calculated as [1–(%TCA soluble radioactivity in the presence of inhibitor)/(% TCA soluble in the absence of inhibitor)]×100%. Inhibition of proteasomal degradation was determined by dividing inhibition by bortezomib by inhibition by ZL3ek.

Values are averages ± SEM of *n* independent experiments for inhibition of protein degradation. Data on viable cells are from Fig. 1B, where errors are shown.

To determine the contribution of total proteasome activity to this process, we used the highly specific proteasome inhibitor ZL3ek [25] at a concentration that blocks 99% of chymotrypsin-like activity and at least 80% of the two other proteasome activities in the majority of cell lines (Table S3). ZL3ek inhibited degradation up to 63% during the first hour of the chase (Table 1). This is similar to the earlier observation that proteasomes degrade 57% of short-lived proteins in exponentially growing fibroblasts [30]. Accordingly, we used the ZL3ek-sensitive fraction of proteins degraded in the first hour as indication of the total proteasome activity so that we could quantify inhibition of proteasomal protein degradation by the different treatments.

**Figure 4:**
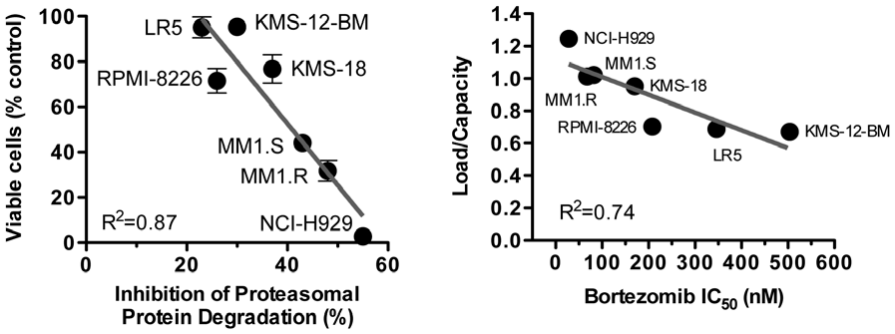
Correlation between load/capacity and sensitivity. A. Plot of viable cells vs. inhibition of protein degradation (from Table 1). B. Plot of load/capacity (from Table 2) vs. IC_50_ (from Figure 1).

1 h pre-treatment with 100 nM bortezomib had dramatically different effects on the degradation of short-lived proteins in different cell lines. In the most sensitive cell lines (NCI-H929), where this concentration reduced the viability by 97%, bortezomib inhibited proteasome-dependent degradation by 56% (Table 1). In the LR5 cell line, there was only 23% inhibition of proteasome-dependent protein breakdown and almost no reduction of viability. Overall there was a very good correlation (r^2^ = 0.87) between the inhibition of protein breakdown and the reduction in number of viable cells (Fig. 4A). Thus, at clinically achievable levels of active site inhibition, degradation of short-lived proteins by proteasomes is inhibited by more than 40% in highly sensitive cell lines but to a lesser extent in resistant cell lines.

These data suggest that the differences in sensitivity can be explained by different inhibition of protein breakdown. If this is the case, then 50% or higher inhibition of proteasomal protein degradation should be observed in resistant cell lines at concentrations that cause 90% reduction in viable cells. Indeed, 900 nM bortezomib inhibited proteasomal degradation of short-lived proteins by 86% in KMS-18 cells, by 57% in RPMI-8226 cells and by 48% in LR5 cells, where it reduced viable cells to 12, 11 and 13% respectively. 2.7 µM bortezomib caused 69% inhibition in KMS-12-BM lines and reduced viable cells to 6% (Table 1). Therefore, bortezomib needs to block at least 50% of protein degradation by proteasomes in order to achieve ∼85% reduction of viable cells.

### Analysis of load/capacity

The surprising finding that similar inhibition of active sites leads to different levels of inhibition of protein degradation is consistent with the load/capacity hypothesis. It postulates that differences in sensitivity can be explained by differences in the ratio of the amount of proteins degraded by the proteasome (load) versus the amount of active proteasomes (capacity) [19]. This hypothesis predicts that similar occupancy of active sites by an inhibitor would cause a stronger effect on protein degradation in cells with higher load/capacity ratio. The variable effect of 100 nM bortezomib on protein breakdown may indicate that load/capacity in bortezomib-sensitive NCI-H929, MM1.R and MM1.S cell lines are higher than in cell lines that are less sensitive to bortezomib and that bortezomib sensitivity is indeed determined by load/capacity ratio. This hypothesis was previously proposed based on the comparison of load/capacity ratios in a limited number (four) of cell lines, treated continuously with proteasome inhibitors [19]. The independent data supporting this hypothesis is that sensitivity of myeloma cells depends on their level of production of immunoglobulins [20], [31]–[33]. To determine whether differences in sensitivity to bortezomib in our panel of seven cell lines can be explained by differences in the load/capacity we decided to determine the load/capacity in all 7 cell lines in the panel and correlate it with IC_50_ after the 1-h treatment.

In order to measure load on the proteasomes (i.e., amount of proteins degraded), it is necessary to determine the amount of protein degradation and the contribution of proteasome to protein turnover. Furthermore, because the rate of protein degradation is normalized to the total amount of radioactivity incorporated in proteins during the pulse, the differences in the amounts of [^3^H]Leu incorporation also have to be accounted for. We found that the cells differed more than three-fold in amounts of radioactive Leu incorporated during a 1-h pulse-labeling (Table 2). The proteasome load was then calculated for each cell line as the amount of protein synthesized (i.e., amount of [^3^H]Leu incorporated)×rate of degradation of short-lived proteins (i.e., amount of [^3^H]Leu released)×percent inhibition of degradation by ZL3ek (Table 2).

**Table 2:** Calculation of the proteasome load/capacity ratio in MM cell lines.

	[^3^H]Leu incorporated	Protein Degradation total			Proteasome Load	Capacity	Load/capacity
Cell Line	(%/h)	As % [^3^H]Leu incorpo-rated/h	As % of [^3^H]Leu used for labeling/h	% inhibition by 10 µM ZL3ek	% total [^3^H]Leu used for labeling/h	Specific activity, pmole/min/cell	
NCI-H929	1.9±0.2	30.0	0.57	61	0.35	0.28±0.03	1.25
MM1.R	1.2±0.1	26.3	0.31	60	0.18	0.18±0.01	1.01
MM1.S	0.7±0.1	26.1	0.19	70	0.14	0.13±0.03	1.02
KMS-18	2.4±0.1	26.3	0.62	62	0.38	0.40±0.01	0.95
RPMI-8226	2.0±0.2	26.2	0.52	62	0.33	0.46±0.04	0.70
LR5	1.2±0.3	23.2	0.27	73	0.20	0.28±0.01	0.69
KMS-12-BM	1.2±0.1	25.7	0.32	54	0.17	0.26±0.02	0.67

Proteasome load = % [^3^H]Leu incorporated in the protein during a 1 h incubation×% of short–lived proteins broken down during first hour of the chase×inhibition by ZL3ek. Capacity = specific activity of the chymotrypsin-like sites. To calculate activity per cells chymotrypsin-like activity was measured in lysates prepared in the whole cell lysis buffer from a defined number of cells. Results for a specific activity are averages ± S.E.M of three biological replicates.

Protein synthesis was measured for 1 h as described in Experimental procedures. Values are averages ± S.E.M of 2–6 independent experiments. % inhibition by ZL3ek is from Table 1.

Load/capacity was calculated by dividing proteasomal degradation by specific chymotrypsin-like activity.

To estimate the relative contribution of long-lived proteins to proteasome load, we metabolically labeled proteins in NCI-H929 and RPMI-8226 cells for 16 h, allowed degradation of short-lived proteins to proceed for 1 h, and then analyzed the breakdown for the subsequent 2 h. The rate of degradation was 5–6% of [^3^H]Leu incorporated in proteins, and near complete inhibition of proteasome led to 50–60% rate decrease (Table 3). Simple calculations described in have allowed us to estimate that the contribution of long-lived proteins to the overall load on the proteasome is 9–10% (Table 3). Therefore, we concluded that most of the load on proteasome comes from short-lived proteins and based all comparisons of the load on the degradation of short-lived proteins.

**Table 3:** Contribution of long-leaved proteins to proteasome load.

	[^3^H]Leu incorporated	Long-Lived Protein Degradation As % of			Proteasome load		LLP contribution to total load^6^
Cell Line	(%/h)^1^	[^3^H]Leu incorporated/h^2^	total [^3^H]Leu used for labeling/h^3^	(% inhibition by ZL3ek)	(% of [^3^H]Leu used for labeling/h)		(%)
					LLP^4^	total^5^	
NCI-H929	1.16	6.4	0.075	51	0.038	0.383	9.9
RPMI-8226	1.13	4.9	0.055	62	0.034	0.359	9.5

1 Calculated as % of [^3^H]Leu incorporated into proteins (i.e., TCA-precipitable) after 16-h labeling/16 h.

2 % Of [^3^H]Leu released into TCA-soluble fraction/h, relative to the total radioactivity incorporated in proteins after 16-h labeling.

3 Long-lived protein degradation (as % of total [^3^H]Leu used for labeling/h) = Long-lived protein degradation (as % of [^3^H]Leu incorporated/h, column 3)×[^3^H]Leu incorporation (%/h, column 2)/100%.

4 Proteasome load from long-lived protein (LLP) = % of total [^3^H]Leu used for labeling/h (column 4)×% inhibition by ZL3ek (column 5)/100%.

5 Proteasome load total = LLP load on proteasome+short-lived proteins load on proteasome (Table 2, column 6).

6 LLP contribution to total load = LLP load on proteasome/total load×100%.

The proteasome capacity was measured as the amount of specific, ZL3ek-inhibitable, chymotrypsin-like activity (the other two activities were proportional to the chymotrypsin-like activity in all cell lines, not shown) in total cell extract (per number of cells lysed). Other studies have compared proteasome amounts by western blotting with antibodies to proteasome subunits [19], but we believe that measuring activity is a more accurate way to measure the capacity of active proteasomes, as quantification by western blot may include inactive proteasomes (e.g., closed gate form of 20S). Finally, dividing load by capacity gave the load/capacity ratio, which varied from 0.67 (KMS-12-BM) to 1.29 (NCI-H929). The resulting load/capacity numbers were then plotted against IC_50_ for the respective cell lines, with a correlation of r^2^ = 0.74 (Fig. 4B). Taken together, these data strongly support a role of the load/capacity ratio in multiple myeloma sensitivity to proteasome inhibitors.

## Discussions

To our knowledge, this study is the first attempt to investigate the effect of bortezomib on myeloma cells in vitro under conditions that resemble in vivo conditions, in which the drug is given as a bolus injection. This study offers several important and novel observations. First, shortening treatment to 1 h reveals greater differences in sensitivity between cell lines than continuous treatment (Fig. 1). Second, myeloma cells differ in the rates at which they undergo apoptosis upon bortezomib treatment (Fig. 2). Third, in the majority of cell lines, clinically achievable inhibition does not reduce cell viability (Fig. 3). Fourth, similar inhibition of active sites results in a markedly different inhibition of protein breakdown in different cell lines (Table 1). Fifth, ∼50% inhibition of proteasomal protein breakdown is needed to reduce cell viability by 90%.

The observation that myeloma cells do not undergo apoptosis upon clinically achievable 85% inhibition of the chymotrypsin-like sites (or perhaps even lower inhibition in the bone marrow [11]) may appear to be contradictory to the 40% response rate to single-agent bortezomib observed clinically [7], [34]. Another paradox is that cells derived from other cancers are as sensitive to bortezomib in vitro as myeloma cells [15] but clinically myeloma is the most bortezomib-responsive malignancy. One possible explanation for these differences is that myelomas are more sensitive to proteasome inhibitors when placed in the bone marrow microenvironment in vivo [35], suggesting that myeloma cell are sensitized to bortezomib in vivo by its microenvironment. This sensitization has not yet been reproduced in vitro as culturing MM cells in the presence of bone marrow stromal cells either had no effect [36] or reduced [37] their sensitivity to bortezomib. In culture, increases in immunoglobulin secretion sensitize myeloma [20], [32] and plasma [33] cells to bortezomib. Conversely, treatment with cycloheximide to inhibit protein synthesis de-sensitizes them to proteasome inhibitors [33]. Lower protein synthesis in established myeloma cell lines may make them less sensitive to bortezomib compared to MM tumors in vivo. In the future it will be interesting to determine whether a patient's response to bortezomib can be predicted based on the sensitivity of freshly-isolated myeloma cells to *ex vivo* pulse treatment with bortezomib.

### Correlation between inhibition of protein degradation and cytotoxicity

The effects of bortezomib on the degradation of short-lived proteins in multiple myeloma cells have not been reported in the literature. This study established that, on average, 60% of short-lived proteins in myeloma cells are degraded by the proteasomes, consistent with earlier findings in fibroblasts [30]. Inhibiting about half of the proteasomal protein degradation results in an 85% decrease in viable cells.

Chymotrypsin-like sites have long been considered the most important sites for protein degradation. However, our earlier work studying degradation of long-lived proteins in HeLa cells has established that inhibition of the chymotrypsin-like sites alone is not sufficient to block protein degradation, and co-inhibition of either caspase-like and trypsin-like sites is usually needed to achieve this [17]. The results of the present study confirm this conclusion. Even in the most sensitive NCI-H929 cell line, only half of proteasomal proteolysis is inhibited upon 95% inhibition of chymotrypsin-like activity and 70% inhibition of caspase-like activity (at 100 nM bortezomib). In RPMI-8226 cells, 95% inhibition of these two sites and 40% inhibition of trypsin-like sites are observed upon 64% inhibition of proteasomal proteolysis (Fig. 3 and Table 1). Thus, inhibition of chymotrypsin-like sites alone is not sufficient to inhibit protein degradation.

### Molecular basis of differences in sensitivity to bortezomib

The most surprising observation of this study was that similar inhibition of active sites (by 100 nM bortezomib) causes different inhibition of protein degradation in different cell lines, and that a higher concentration of bortezomib is needed to achieve 50% inhibition of proteasomal proteolysis in less sensitive cell lines. Although some difference was observed in the recovery of proteasome activity after removing bortezomib, the differences in the first hour after withdrawing the drug were much smaller than the differences in the inhibition of protein degradation. This observation, together with the correlation between load/capacity and IC_50_ is consistent with the hypothesis that the sensitivity of myeloma cells to bortezomib depends on the ratio of proteasome load to proteasome capacity [19].

While this manuscript was under review, it was reported that treatment with a proteasome inhibitor-induced apoptosis is the consequence of amino acid depletion from culture media of Drosophila cells, NIH3T3 mouse fibroblasts, human embryonal kidney 293T and human cervical carcinoma HeLa cells, followed by stress response and apoptosis [38]. Although the conclusions about mechanisms of proteasome-inhibitor induced apoptosis in these non-secretory cells are different from ones obtained in this study, these discrepancies raise the possibility that proteasome inhibitors activate different apoptosis mechanisms in non-secretory cells expected to have lower load on proteasome.

In summary, this study illustrates the importance of using clinically relevant concentrations and treatment durations of pharmacologic agents during in vitro experiments. This approach revealed important insights into the mechanism of differential sensitivity of myeloma cells to the proteasome inhibitor bortezomib. Using this approach, we discovered that similar inhibition of active sites across cell lines does not necessarily result in similar inhibition of protein degradation.

## Supporting Information

Table S1 **Three washes are sufficient to remove free [^3^H]Leu from the cells during pulse-chase experiments.** After pulsing NCI-H929 cells for 1 h with 10 µCi/ml [^3^H]Leu, cells were washed four times with media containing 2.5× cold Leu, and amounts of radioactivity in each sample determined on the scintillation counter.
(DOC)

Table S2 **Treatment of cells with proteasome inhibitors during the 1-h pulse does not affect incorporation of [^3^H]Leu.** The rates of [^3^H]Leu incorporation into 10% TCA insoluble fraction in the presence and absence of inhibitors was determined as described in Experimental Procedures.
(DOC)

Table S3 **Effect of ZL3-ek on proteasome activity.** MM cells were treated with 10 µM ZL3ek for 1 h, and after removal of the inhibitor proteasome activities were measured with Proteasome Glo assay as described [17]. Mock-treated cells served as controls. Values are averages (± S.E.M) of 2–3 independent experiments (biological replicates).
(DOC)

Figure S1 **Treatment with bortezomib does not up-regulate proteasome.** Cells were treated with bortezomib, and then cultured in the absence of inhibitors. At times indicated, a fraction of cells was harvested, and lysed in the whole cell lysis buffer. A. Western blot analysis using anti-Rpt5, α6, and α5 antibodies (Enzo). The double Rpt5 band most likely is the consequence of post-translational modification. B. Quantification of western blots in panel A using Odyssey fluorescent scanner. Data is mean±S.E.M. of two independent experiments.
(TIF)
