# Supplementary material for: Molecular Basis of Differential Sensitivity of Myeloma Cells to Clinically Relevant Bolus Treatment with Bortezomib
Source: PLoS One. 2013 Feb 27;8(2):e56132. doi: 10.1371/journal.pone.0056132 (PMC3584083; doi:10.1371/journal.pone.0056132)
Supplement: Table S1 — Three washes are sufficient to remove free [3H]Leu from the cells during pulse-chase experiments. After pulsing NCI-H929 cells for 1 h with 10 µCi/ml [3H]Leu, cells were washed four times with media containing 2.5× cold Leu, and amounts of radioactivity in each sample determined on the scintillation counter. (DOC) [file pone.0056132.s001.doc]

**Table S1. Three washes are sufficient to remove free [^3^H]Leu from the cells during pulse-chase experiments.**

| Sample | cpm |
| --- | --- |
| Incorporate in proteins | 2,986±140 |
| Unincorporated | 104,459 ± 1,154 |
| 1st Wash | 2,155 |
| 2nd Wash | 188 |
| 3rd Wash | 70 |
| 4th Wash | 51 |
| blank | 40 |
