# Supplementary material for: Molecular Basis of Differential Sensitivity of Myeloma Cells to Clinically Relevant Bolus Treatment with Bortezomib
Source: PLoS One. 2013 Feb 27;8(2):e56132. doi: 10.1371/journal.pone.0056132 (PMC3584083; doi:10.1371/journal.pone.0056132)
Supplement: Table S2 — Treatment of cells with proteasome inhibitors during the 1-h pulse does not affect incorporation of [3H]Leu. The rates of [3H]Leu incorporation into 10% TCA insoluble fraction in the presence and absence of inhibitors was determined as described in Experimental Procedures. (DOC) [file pone.0056132.s002.doc]

**Table S2. Treatment of cells with proteasome inhibitors during the 1-h pulse does not affect incorporation of** **[^3^H]Leu.**

| Cell line | Inhibitor | (μM) | % Inhibition of Incorporation, (Average ± S.E.M.) |
| --- | --- | --- | --- |
| NCI-H929 | ZL3ek | 10 | -4.3 ± 4.2 |
|  | bortezomib | 0.1 | 0.5 ± 3.2 |
| MM1.R | ZL3ek | 10 | -0.3 ± 4.5 |
|  | bortezomib | 0.1 | -7.8 ± 2.7 |
| KMS-18 | ZL3ek | 10 | -1.0 ± 2.3 |
|  | ALLN | 50 | -1.3 ± 3.3 |
|  | bortezomib | 0.1 | -4.7 ± 3.1 |
|  | bortezomib | 0.9 | -3.3 ± 4.9 |
| KMS-12-BM | ZL3ek | 10 | 0.8 ± 6.1 |
|  | ALLN | 50 | 2.0 ± 3.1 |
|  | bortezomib | 0.1 | -3.7 ± 2.9 |
|  | bortezomib | 2.7 | -5.6 ± 3.0 |
| RPMI-8226 | ZL3ek | 10 | -2.1 ± 2.9 |
|  | ALLN | 50 | -2.8 ± 5.4 |
|  | bortezomib | 0.1 | -4.1 ± 6.0 |
|  | bortezomib | 0.9 | -7.2 ± 3.6 |
| MM1.S | ZL3ek | 10 | 5.4 ± 7.6 |
| LR5 | ZL3ek | 10 | 1.6 ± 4.0 |
