# Supplementary material for: Molecular Basis of Differential Sensitivity of Myeloma Cells to Clinically Relevant Bolus Treatment with Bortezomib
Source: PLoS One. 2013 Feb 27;8(2):e56132. doi: 10.1371/journal.pone.0056132 (PMC3584083; doi:10.1371/journal.pone.0056132)
Supplement: Table S3 — Effect of ZL3-ek on proteasome activity. MM cells were treated with 10 µM ZL3ek for 1 h, and after removal of the inhibitor proteasome activities were measured with Proteasome Glo assay as described [17]. Mock-treated cells served as controls. Values are averages (± S.E.M) of 2–3 independent experiments (biological replicates). (DOC) [file pone.0056132.s003.doc]

**Table S3. Effect of ZL3-ek on proteasome activity.**

| **MM Cell Line** | **Chymotrypsin-like** | **Caspase-like** | **Trypsin-like** |
| --- | --- | --- | --- |
|  | Inhibition (% control) | | |
| H929 | 98 ± 2 | 78 ± 2 | 77 ± 2 |
| RPMI-8226 | 97 ± 2 | 76 ± 7 | 53 ± 6 |
| MM1.R | 99 ± 3 | 94 ± 2 | 90± 0.3 |
| MM1.S | 100 ± 0.9 | 93 ± 3 | 94 ± 7 |
| LR5 | 99 ± 1 | 85 ± 0.8 | 76 ± 3 |
| KMS-12-BM | 99 ± 2 | 88 ± 3 | 91 ± 9 |
| KMS-18 | 97 ± 2 | 69 ± 13 | 67 ± 6 |
