# Supplementary figures and images for: Molecular Basis of Differential Sensitivity of Myeloma Cells to Clinically Relevant Bolus Treatment with Bortezomib
Source: PLoS One. 2013 Feb 27;8(2):e56132. doi: 10.1371/journal.pone.0056132 (PMC3584083; doi:10.1371/journal.pone.0056132)

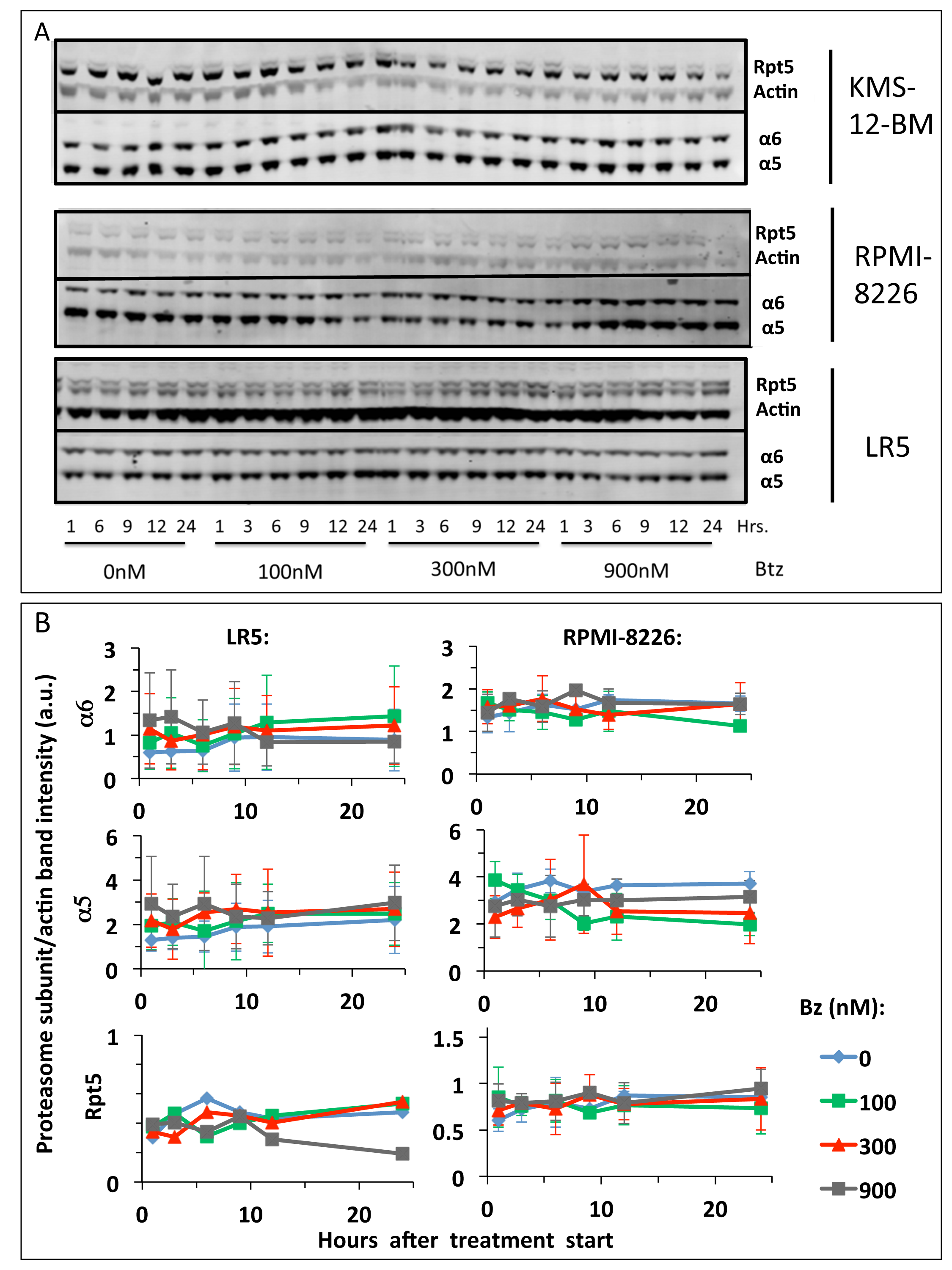

Supplement: Figure S1 — Treatment with bortezomib does not up-regulate proteasome. Cells were treated with bortezomib, and then cultured in the absence of inhibitors. At times indicated, a fraction of cells was harvested, and lysed in the whole cell lysis buffer. A. Western blot analysis using anti-Rpt5, α6, and α5 antibodies (Enzo). The double Rpt5 band most likely is the consequence of post-translational modification. B. Quantification of western blots in panel A using Odyssey fluorescent scanner. Data is mean±S.E.M. of two independent experiments. (TIF) [file pone.0056132.s004.tif]
